# Effectiveness and safety of ShenXiong glucose injection for acute ischemic stroke: a systematic review and GRADE approach

**DOI:** 10.1186/s12906-016-1038-8

**Published:** 2016-02-19

**Authors:** Xue-ting Liu, Peng-wei Ren, Le Peng, De-ying Kang, Tian-le Zhang, Shu Wen, Qi Hong, Wen-jie Yang

**Affiliations:** Department of Evidence-based Medicine and Clinical Epidemiology, West China Hospital, Sichuan University, Chengdu, 610041 P. R. China

**Keywords:** ShenXiong glucose injection, Acute ischemic stroke (AIS), Systematic review (SR), GRADE

## Abstract

**Background:**

To appraise critically whether published trials of ShenXiong glucose injection for patients with acute ischemic stroke (AIS) are of sufficient quality, and in addition to rate the quality of evidence by using the GRADE approach (grading of recommendations, assessment, development, and evaluation, GRADE).

**Methods:**

A literature search was performed in the Cochrane Library, MEDLINE, EMBASE, CBM, Chinese TCM (traditional Chinese medicine) Database, CNKI, VIP, WanFang Databases until January 2015. The limits were patients with AIS and randomized controlled trials (RCTs) or quasi-RCTs. Studies by which patients suffering intracerebral haemorrhage were excluded.

**Results:**

Twelve studies fulfilled the inclusion criteria. We found significant benefits of ShenXiong glucose injection compared with conventional treatment in improving activities of daily living function at 4 weeks (MD = 34.12, 95 % CI: 29.07, 39.17), neurological function deficit at 2 weeks (MD = −5.39, 95 % CI: −6.90, −3.87), 4 weeks (MD = −5.16, 95 % CI: −6.49, −3.83), and clinical effects at 4 weeks (RR = 1.17, 95 % CI: 1.10, 1.24). No trials reported the effects of ShenXiong glucose injection on the risk of early, deterioration, or quality of life. No adverse events were reported within the whole follow-up period.

**Conclusions:**

The use of ShenXiong glucose injection may improve rehabilitation for patients with acute ischemic stroke, however, as the GRADE approach indicated low to moderate quality of available evidence as well as insufficient information about harm and patients preference, the recommendations were not provided for ShenXiong glucose injection taking as a therapeutic intervention to patients with acute ischemic stroke.

**Electronic supplementary material:**

The online version of this article (doi:10.1186/s12906-016-1038-8) contains supplementary material, which is available to authorized users.

## Background

Stroke occurs when the blood supply to the brain is interrupted or reduced, which may be caused by blocked arteries or leaking and bursting of blood vessels [1]. This deprives the brain of the supply of oxygen and nutrients, causing damage to the brain tissue [2]. Being one of the most severe neurological diseases, stroke often leads to death or gross physical impairment or disability [2], and the morbidity of stroke continues to rise with an annual increase of 8.1 %. Although the incidence of stroke is declining in many developed countries, the absolute number of stroke patients continues to enlarge because of the ageing population [3]. Of those, acute ischemic stroke as the dominant type of stroke, account for 85 % of all strokes [4].

Currently, rapid assessment and early intervention for patients suffering an acute ischemic stroke is of top priority [5, 6]. There is as yet no routine, effective, generally accepted, specific treatment for AIS, except for aspirin [7] and anti-thrombolysis with tissue plasminogen activator approved by FDA of US is administered in the setting of AIS within 4.5 h from symptom-onset [8–10]. Therefore, it is necessary to test other promising therapeutic approaches to acute ischemic stroke [11, 12]. The lack of effective and widely applicable pharmacological treatments for acute ischemic stroke patients leads to a growing interest in Chinese traditional medicine [13, 14], for which extensive observational and anecdotal experience has accumulated over the past one thousand years [14, 15]. Of those, Chinese herbal medicine is the most addressed in China, US and European Union [12, 13]. In China, a series of optional treatments for treating AIS are available. Traditional Chinese medicine have proven to be effective complementary intervention for AIS, of which Chinese herb products are still prevailing well and make a significant difference [12, 14, 15]. The overall treatment concept for Chinese traditional medicine is different from Western medicine, and herb medicines were always used to treat acute ischemic stroke. ShenXiong glucose injection is one of the most widely used Chinese herb medicine.

ShenXiong glucose injection (also named as Danshen-Chuanxiongqin injection) has properties that might be effective in acute ischemic stroke [16], it is on the national essential drug list of China as a new drug for cardio-cerebral vascular diseases. ShenXiong glucose injection is a compound preparation, made up of Salvia miltiorrhiza and Ligustrazine [16]. Of those, fifteen kinds of biologically active substances have been extracted from the dried roots of Salvia miltiorrhiza, a traditional medical herb known as Danshen, among which tanshinone, isotanshinone and hydroxy-tanshinone are the important ingredients [16, 17]. Various studies suggested that Danshen could decrease intracranial pressure by decreasing overall water content of the brain and cerebrospinal fluid volume, and by reducing blood volume due to vasoconstriction [18, 19]. DanShen may also improve cerebral perfusion by decreasing viscosity. It is reported to increase coronary blood flow, suppress thromboxane formation, decrease cerebral oedema, infarct size and neurological deficit [19]. It is always used either alone or in combination with other herbal ingredients for patients with coronary heart diseases, hyperlipidemia, cerebrovascular diseases. In both China and other countries like US, such findings come either from biomedical studies [19] or from clinical trials [19, 20]. Ligustrazine, a bioactive ingredient extracted from Chuanxiong (Rhizoma Chuanxiong), was also proved to have beneficial effect on cerebrovascular diseases in pharmacological studies [21]. ChuanXiong could improve brain microcirculation by preventing thrombus formation and platelet aggregation and blood viscosity in order that it might have significant beneficial effects in the treatment of acute ischemic stroke [21]. As the compound of Salvia miltiorrhiza and ligustrazine, ShenXiong glucose injection has been widely accepted as a standard treatment for acute ischemic stroke in China for over 15 years [16]. Its mechanism is associated with exciting histamine receptors, improving neuron function and reducing serum total cholesterol-triglyceride levels, increasing serum high density lipoprotein levels, and enhancing tissue plasminogen activator activity. Moreover, ShenXiong glucose injection can prevent free radical injury from ischemic brain tissue by reducing lipid peroxide [16].

Currently there are a large number of studies of the clinical efficacy of ShenXiong glucose injection in acute ischemic stroke published in the global, especially in China. However, those clinical trials have not been reviewed systematically at present, whether the existing evidence is of sufficient scientifically rigorous and whether ShenXiong glucose injection can be recommended for routine use based on current evidence is still uncertain. To make specific recommendations for further research into the effectiveness and safety of ShenXiong glucose injection for patients with acute ischemic stroke, we carry out a systematic review and GRADE approach in twofold: (i) to systematically review all the randomized and quasi-randomized controlled trials of ShenXiong glucose injection for acute ischemic stroke; (ii) in addition, to evaluate the quality of current evidence by the use of GRADE system, and to provide the best available evidence for clinical practice.

## Methods

### Criteria for considering studies for this review and GRADE approach

#### Type of studies

We included all randomized controlled trials (RCTs), and quasi-randomized controlled trials (trials that used a non-random method of treatment allocation, e.g. hospital number, date of birth or day of the week), either published or unpublished.

#### Types of participants

Trials involving patients of any age or sex with apparent acute ischemic stroke (excluding intracerebral hemorrhage) were eligible. Stroke must be diagnosed in accordance with the diagnostic criteria of neuro-imaging verification of pathos-logical alterations in the brain, with computed tomography (CT) or Magnetic Resonance Imaging (MRI). Of these criteria, “Key Points for Diagnosing Cerebrovascular Diseases” and “Stroke diagnosis curative standard” modified in the 4th National Cerebrovascular Disease Seminar by the China Medical Society in 1995 [22] and “Guidelines of diagnosis and treatment of Chinese acute ischemic stroke” in 2010 [23], were adapted.

#### Type of interventions

Regardless of frequency, intensity or the duration of treatment, either ShenXiong glucose injection versus placebo or ShenXiong injection plus conventional treatments versus conventional treatments alone were included in this review and GRADE approach.

Any other routine treatments except ShenXiong glucose injection, such as antihypertensive, anti-platelet aggregation and other symptomatic treatments if necessary, were defined as conventional treatment, and all participants either in experimental group or in controlled group would receive same conventional treatments during whole trial period.

#### Type of outcome measures

The primary outcome is death or dependency at the end of scheduled follow-up. Dependency is defined as severely dependent on others in activity of daily living, based on the definition of the Barthel Index scores 60 or less, or an Oxford handicap grade 3 to 6.

Secondary outcomes were: (1) Changes of neurological deficit after ShenXiong glucose injection treatment and at the end of scheduled follow up. The measures could concentrate on specific impairment (for instance, Motricity Index, Motor Assessment Scale) or global neurological deficit (for instance, the National Institute of Health Stroke Scale or the Neurological Function Deficit Score). The clinical neurological impairment score of stroke patients is measured by the National Institute of Health Stroke Scale (NIHSS) or the Neurological Function Deficit Score (NFDS) . The neurological function deficit score changes, such as NFDS reduction rate over 18 % was taken as curative effect standard [24]; (2) Quality of life (QOL) at the end of follow up, which is measured by the Nottingham Health Profiles or Spiter Quality of Life Index; (3) Adverse events including bleeding, dizziness, vomiting, allergic reaction, or other adverse events caused by ShenXiong glucose injection. The number of patients developing at least one adverse event listed above was estimated.

### Search strategies

#### Electronic Search

We searched the Cochrane Central Register of Controlled Trials (CENTRAL, Ovid, from 1991 to January 2015), MEDLINE (PubMed, from 1966 to January 2015), EMBASE (Ovid, from 1966 to January 2015), CBM (Chinese Biomedicine Database, from 1978 to January 2015), Chinese TCM Database (from 1949 to January 2015), CNKI (China National Knowledge Infrastructure, until January 2015), VIP (Chinese Scientific Journal Database from 1989 to January 2015), WanFang Database (from 1998 to January 2015). The key words comprise DanshenChuanxiongqin, Danshen-Chuanxiongqin, ShenXiong glucose injection, Dan Xiong and ShenXiong injection were used as English and corresponding Chinese search terms to identify studies from aforementioned databases. Reference lists of all included studies were also searched for publications that meet the inclusion criteria.

### Searching other resources

In order to search all the related studies, we also hand-searched the following relevant journals of TCM (the last issues were set at January 2015): World Journal of Integrated Traditional and Western Medicine, Chinese Journal of Integrative Medicine, Journal of Chinese Integrative Medicine, Journal of Traditional Chinese Medicine and Journal of Beijing University of Traditional Chinese Medicine.

### Data collection and analysis

#### Selection of studies

Clinical trials were identified by screening the titles and abstracts of targets by two independent reviewers (XL, LP), and any disagreements were resolved through discussion and consultation with a third reviewer (DK).

### Data extraction and management

Two reviewers (XL, LP) independently extracted data from studies that met the inclusion criteria using a pre-designed form, and disagreements were resolved by discussing with the third reviewer (DK). We planned to retrieve the following data: methods (study design and execution, method of randomization), participant characteristics (number of patients, age, sex, inclusion criteria, method of diagnosis), interventions (description of interventions given to each treatment group including, with or without other combined treatment), outcomes, methods of analysis (intention-to-treatment analysis or per-protocol analysis, or both) and statistical methods used.

### Assessment of risk of bias in included studies

Two reviewers (XL, LP) independently assessed the methodological quality of each included studies using the “risk of bias” assessment tool outlined in the Cochrane Handbook for Systematic Reviews of interventions (Version 5.1.0) [25]. Any disagreements were resolved by discussion with the third reviewer (DK).

### Statistical analysis

The Cochrane Review Manager software, RevMan 5.1 [26], was used to calculate treatment effects across trials. For discontinuous data, we used relative risk (RR) with the corresponding 95 % confidence interval (CI) as the measure of effect. For continuous data, the measure of the treatment difference for any outcome would be the mean difference (MD) when the pooled trials using the same rating scale or test, otherwise, the standardized mean difference (SMD) with 95 % confidence interval (CI) would be used. A Chi-square based test of homogeneity was performed using Cochrane Q statistic [27] and I^2^. If *P* value less than 0.10 or I^2^ value exceed 50 % substantial, indicated heterogeneity among trial results significantly, we would carry out a subgroup analysis to explore the sources of heterogeneity, including clinical heterogeneity, such as the route of administration, time and dose interval of start of treatment, type of intervention, as well as the methodological heterogeneity, such as design type of the trials, the risk bias of happening and so on. If I^2^ was less than or equal to 50 % we would use a fixed-effect meta-analysis, otherwise, if the underline true effect being assumed to follow up a normal distribution, we would use a random-effects model (in which case the confidence intervals will be broader than those of a fixed-effect model). Sensitivity analysis was carried out by using the leave-one-out approach to explore the influence of heterogeneity, to determine how robust the results of analysis were. Funnel plots and Egger analysis were used to assess the existence of publication bias, if possible.

Besides, in the process of interpreting results and coming to conclusions, we planned to use GRADE approach (grading of recommendation assessment, development, and evaluation, GRADE) to evaluate the quality of evidence. This approach is a systematic method for evaluating quality of evidence and formulating strength of recommendation if sufficient information is available [28]. Firstly, we subsequently classified the importance of each outcome. Three categories of outcome were specified based on their importance (median score of 1 to 9). Then the quality of evidence is divided into four categories: high, moderate, low, and very low. The GRADE approach defines clearly the methodological criteria by which evidence can be upgrade or downgrade and the justification for the strength of recommendation being formulated as “strong” or “weak”. Critical and important outcomes were used for decision making and were included in the evidence profile and summary of Findings Tables [29].

## Results

### Literature Search

**Fig. 1:**
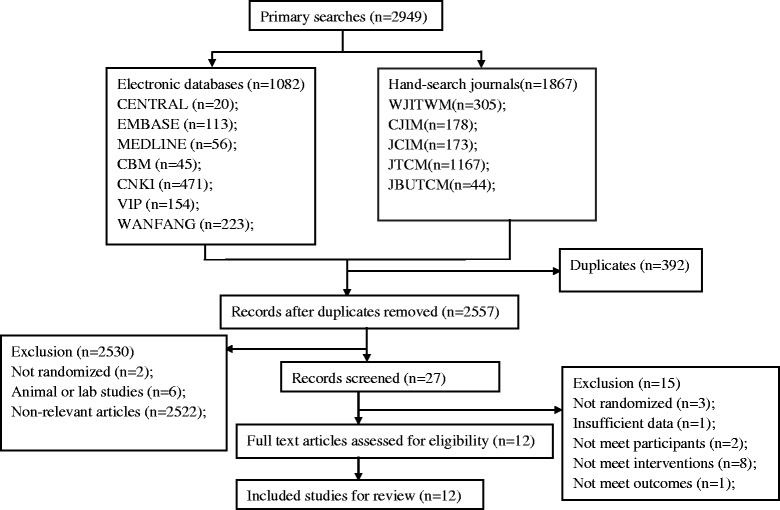
Flow chart of study selection. Abbreviations: CBM, Chinese Biomedical Literature Database; CNKI: China National Knowledge Infrastructure; VIP: Chinese Scientific Journals Database; WJITWM: World Journal of Integrated Traditional and Western Medicine; CJIM: Chinese Journal of Integrative Medicine; JCIM: Journal of Chinese Integrative Medicine; JTCM: Journal of Traditional Chinese Medicine; JBUTCM: Journal of Beijing University of Traditional Chinese Medicine

A flow diagram of study selection is shown in Fig. 1. After initially identifying 2949 articles, 12 studies that met the inclusion criteria were included for this review and GRADE approach (Table 1).

**Table 1:** Characteristics of included studies

First author	Number of patients (n)		Age (years, Mean ± SD)		Diagnostic criteria	Onset (days)	Baseline NFDS (Mean ± SD)		Dose of Experiment	Conventional measure (CM)	Setting
	E	C	E	C			E	C			
Wang 2014 [32]	35	35	67.24 ± 4.38	68.15 ± 5.0	CT,MRI	≤3d	18.15 ± 8.14	17.96 ± 8.02	Danshen Ligustrazine 10 ml/d + 250 ml glucose + CM	antihypertensive, anti platelet aggregation	Inpatients
Liu 2014 [30]	71	71	53.1 ± 1.7	49.7 ± 2.1	CT,MRI	≤3d	22.49 ± 1.21	23.18 ± 1.74	Danshen Ligustrazine 13–15 ml/d + 250 ml glucose + CM	antihypertensive, reduce ICP	Inpatients
Liu 2011 [39]	34	33	41–79	40–80	CT,MRI	≤3d	17.2 ± 2.0	16.7 ± 3.2	Danshen Ligustrazine 10 ml/d + 250 ml glucose + CM	anticoagulation, nutrition brain cells	Inpatients
Zhou 2013 [37]	50	50	54.2 ± 4.1	54.2 ± 4.1	NR	≤3d	NR	NR	Danshen Ligustrazine 10 ml/d + 250 ml glucose + CM	Anti-platelet aggregation, reduce ICP	Inpatients
Huang 2012 [40]	38	30	64.3 ± 5.6	66.3 ± 5.3	CT,MRI	NR	NR	NR	ShenXiong glucose 200 ml plus CM	nutrition brain cells, reduce ICP	Inpatients
Zhou 2011 [34]	40	40	42–75	42–75	CT,MRI	NR	22 ± 10	23 ± 10	ShenXiong glucose 200 ml plus CM	antihypertensive, reduce ICP	Inpatients
Shan 2013 [31]	30	30	65	64	CT,MRI	≤3d	22.50 ± 8.03	21.65 ± 6.2	ShenXiong glucose 200 ml plus CM	antihypertensive, anti platelet aggregation	Inpatients
Zhe 2011 [33]	30	31	57.34 ± 8.97	58.26 ± 7.48	CT,MRI	≤2d	24.63 ± 9.28	23.68 ± 7.89	ShenXiong glucose 200 ml plus CM	nutrition brain cells, reduce ICP, anti-platelet aggregation	Inpatients
Ling 2013 [41]	40	40	56.3 ± 12.5	58.3 ± 11.8	CT,MRI	≤3d	NR	NR	ShenXiong 10 ml/d + 250 ml glucose + CM	anti platelet aggregation, nutrition brain cells	Inpatients
Guan 2011 [42]	46	40	63 ± 3	63 ± 3	CT,MRI	NR	NR	NR	ShenXiong glucose 200 ml plus CM	reduce ICP, nutrition brain cells	Inpatients
Nie 2013 [35]	35	35	55–71	54–70	CT,MRI	NR	25.63 ± 9.08	24.68 ± 7.59	ShenXiong glucose 200 ml plus CM	anti platelet aggregation, nutrition brain cells	Inpatients
Lin 2014 [36]	43	43	55.6 ± 9.31	52.8 ± 8.72	CT,MRI	≤3d	NR	NR	ShenXiong 10 ml/d + 250 ml glucose + CM	anti-hypertensive, anti-platelet aggregation	Inpatients

Notes: *SD* standard deviation, *NR* not report, *NFDS* neurological function deficit score, *CM* Conventional Measure, *CT* Computed Tomography, *MRI* Magnetic Resonance Imaging, *E* experiment group, *C* control group

### Study characteristics and quality assessment

Characteristics of the 12 trials included in this review were summarized in Table 1, the number of participants that received ShenXiong glucose injection treatment ranged from 30 to 71 (total 494), and the number of participants in the control groups ranged from 30 to 71 (total 480). In 12 studies, ShenXiong glucose injection plus conventional therapy was administered in experimental group, the dose of Danshen-Chuanxiongqin ranges from 10 ml/d to 15 ml/d, and the duration of treatment ranged from 2 to 4 weeks.

The results of the quality assessment of the included studies are shown in Table 2. Two studies [30, 31] had low selection bias (allocating group by using random number table), two studies [32, 33] were quasi-randomized trials, other 8 trials reported “randomly allocating” participants, but not stated the method of randomization. Possible performance bias from inappropriate blinding of participants and personnel might be present in 12 studies.

**Table 2:** Assessment of risk of bias in included studies

Study	Random Sequence Generation	Allocation Concealment	Blinding of Participants and personnel	Blinding of Outcome assessment	Incomplete Outcome Data	Selective Reporting	Other source of bias
Wang 2014 [32]	sequence of entering the group	Unclear	Unclear	Unclear	Yes	Yes	Unclear
Liu 2014 [30]	random number table	Unclear	Unclear	Unclear	No	Unclear	Unclear
Liu 2011 [39]	unclear	Unclear	Unclear	Unclear	Yes	No	Unclear
Zhou 2013 [37]	unclear	Unclear	Unclear	Unclear	No	Yes	Unclear
Huang 2012 [40]	unclear	Unclear	Unclear	Unclear	Yes	Unclear	Unclear
Zhou 2011 [34]	unclear	Unclear	Unclear	Unclear	Yes	Yes	Unclear
Shan 2013 [31]	random number table	Unclear	Unclear	Unclear	Yes	Yes	Unclear
Zhe 2011 [33]	sequence of entering the group	Unclear	Unclear	Unclear	Yes	Yes	Unclear
Ling 2013 [41]	unclear	Unclear	Unclear	Unclear	Yes	Unclear	Unclear
Guan 2011 [42]	unclear	Unclear	Unclear	Unclear	No	Unclear	Unclear
Nie 2013 [35]	unclear	Unclear	Unclear	Unclear	Yes	Unclear	Unclear
Lin 2014 [36]	unclear	Unclear	Unclear	Unclear	Yes	Unclear	Unclear

Annotation: Yes = low risk of bias; No = high risk of bias; unclear = uncertain risk of bias

### Effects of interventions

#### Death or dependency at the end of scheduled follow up period

No deaths were reported within the period of treatment in 12 trials. This may mean that only mild strokes were included in the trials or that death occurred were not reported by trialists.

**Fig. 2:**
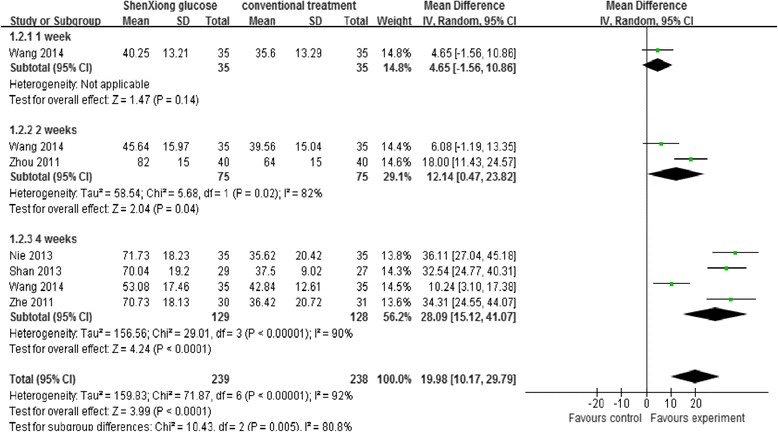
A random effects Meta-analysis on improvement of activities of daily living function of ShenXiong glucose injection

A measure of activities of daily living function was assessed in five [31–35] of the included trials. Barthel index scores was measured at 1 week in only one trial [32], 2 weeks in two studies [32, 34], and 4 weeks on treatment in four studies [31–33, 35]. All of these trials reported sufficient information to allow for inclusion into a meta-analysis. Wang 2014 reported no significant difference on Barthel index score in patients randomized to ShenXiong glucose injection group as compared with control group, by approximately 7 days (MD = 4.65, *P* = 0.14, 95 % CI: −1.56, 10.86); Two studies [32, 34] reported Barthel index score at 14 days, however, a pooled analysis was not performed since severe heterogeneity were detected between the trials (*P* = 0.02, I^2^ = 82 %), which was possibly due to differences in outcome measurements used and types of treatment. The trial by Zhou 2011 reported that there was significant difference in the Barthel index scores at 14 days in the ShenXiong glucose injection group as compared with control group (MD = 18.00, 95 % CI: 11.43, 24.57). Trial by Wang 2014 reported no significant difference in the daily activities score at four weeks between the treatment groups (MD = 6.08, 95 % CI: −1.19, 13.35), but the confidence interval was very wide and included clinically significant effects in both directions (Fig. 2).

**Fig. 3:**
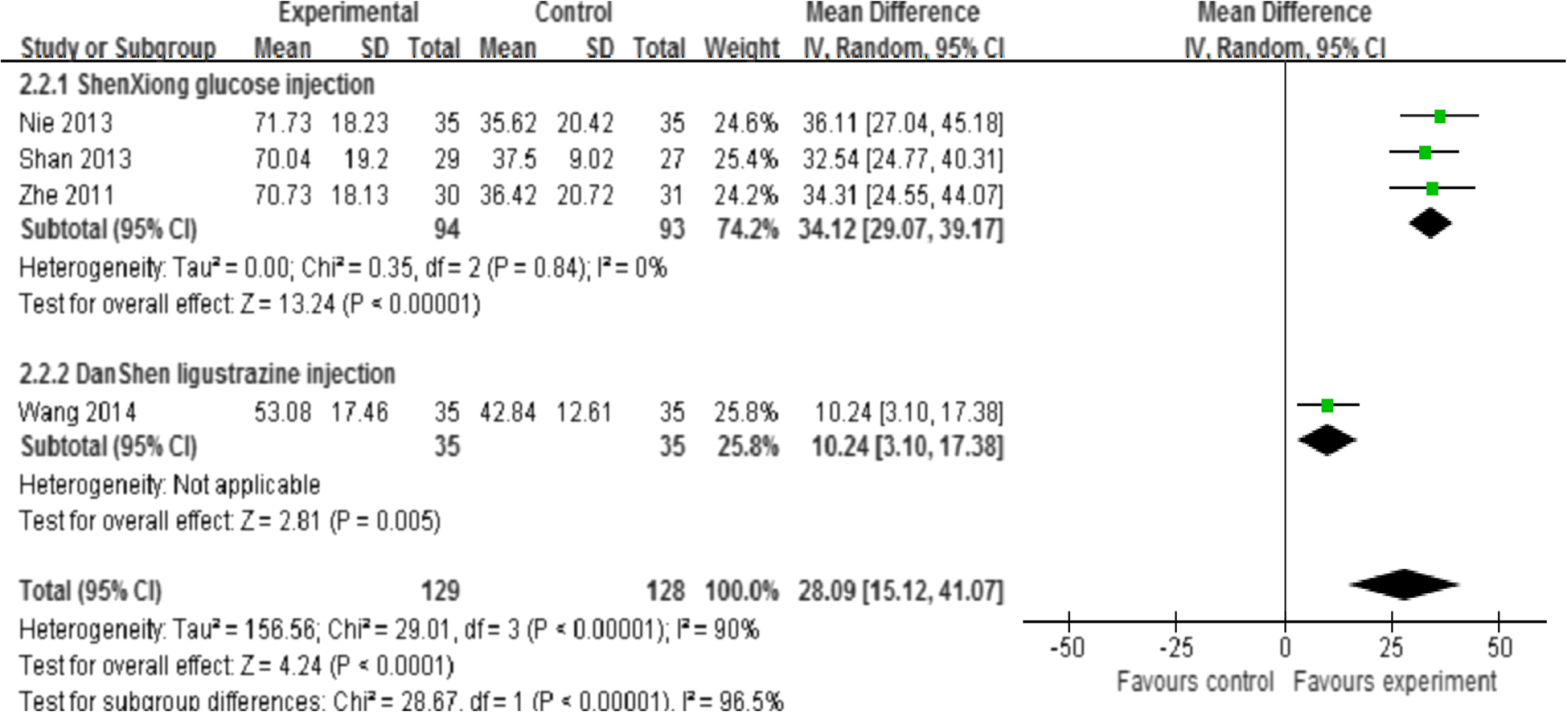
Subgroup analysis. ShenXiong glucose injection vs DanShen ligustrazine injection; Outcome: Improvement of activities of daily living function at 4 weeks follow-up

Four studies [31–33, 35] also assessed Barthel index scores at 28 days of treatment, however a pooled analysis was not performed since severe heterogeneity were detected among the trials (I^2^ = 90 %), which was possibly due to the differences in term of outcome measurements used and types of treatment. All of these trials reported higher Barthel scores at 28 days for the ShenXiong glucose injection group as compared with control group. We performed a subgroup analysis based on the treatment type (ShenXiong glucose injection [31, 33, 35] and Danshen ligustrazine injection [32]). There were significant difference in improvement of activities of daily living function at 28 days in the ShenXiong glucose injection group compared with control group (MD = 34.12, 95 % CI: 29.07, 39.17); as well as in the Danshen ligustrazine injection group compared with control group (MD = 10.24, 95 % CI: 3.10, 17.38) (Fig. 3).

### Improvement of neurological function deficit after treatment and at the end of scheduled follow up

**Fig. 4:**
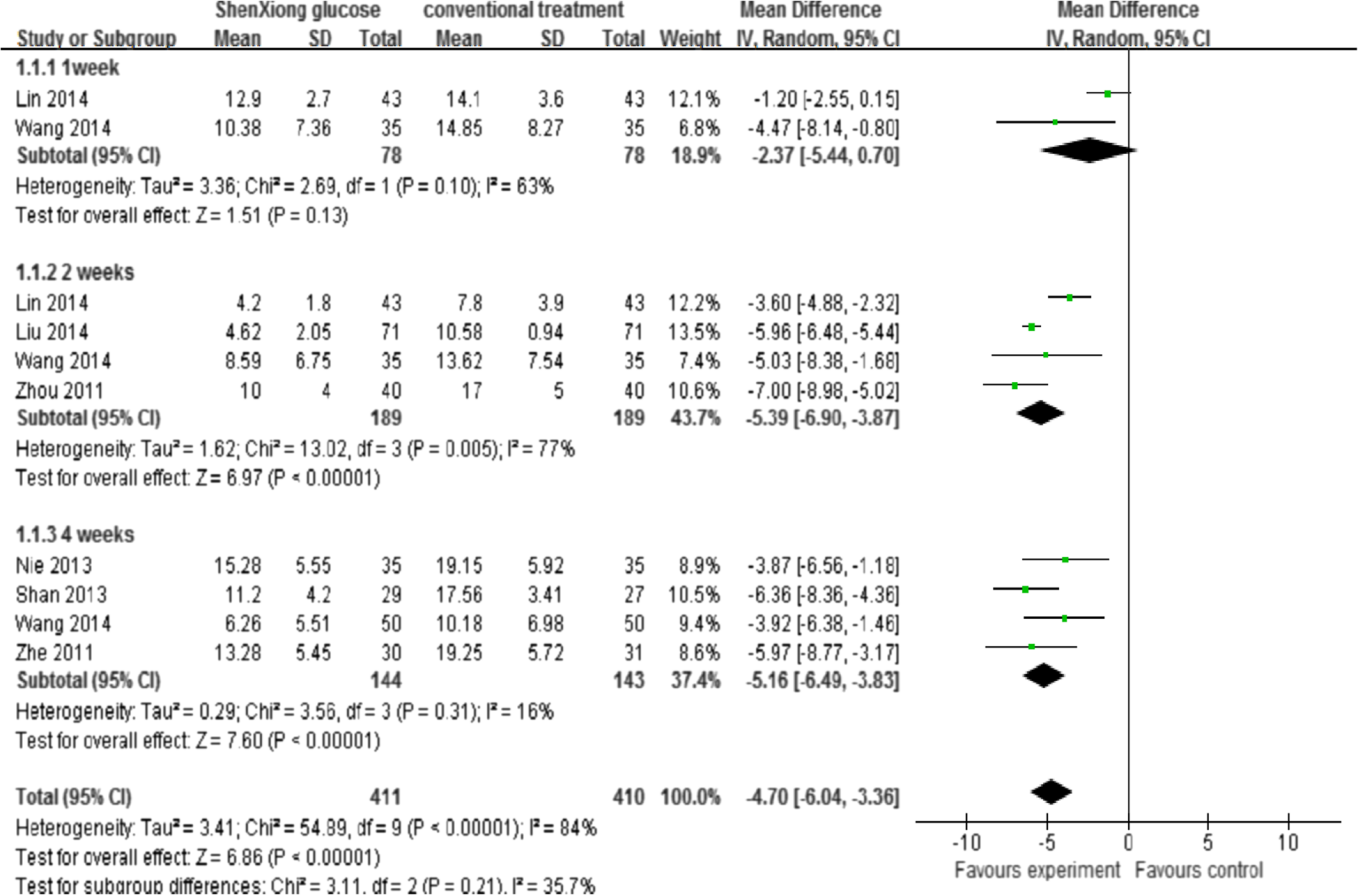
A random effects Meta-analysis on neurological function deficit score of ShenXiong glucose injection

Seven trials [30–36] with a total of 821 patients measured improvement of neurological function deficit after ShenXiong glucose injection treatment by using continuous approaches, such as changes of neurological deficit score. There was significant heterogeneity between studies [30, 32] (Chi-squared *P* = 0.10, I^2^ = 63 %), which may be explained by the differences in outcome measurements used and types of treatment, therefore, the overall estimate of treatment effect should be interpreted with caution, although no significantly improvement of neurological impairment (MD for neurological improvement with ShenXiong glucose injection −2.37, 95 % CI −5.44 to 0.70) were observed at 7 days in the ShenXiong glucose injection group compared with control group (Fig. 4).

**Fig. 5:**
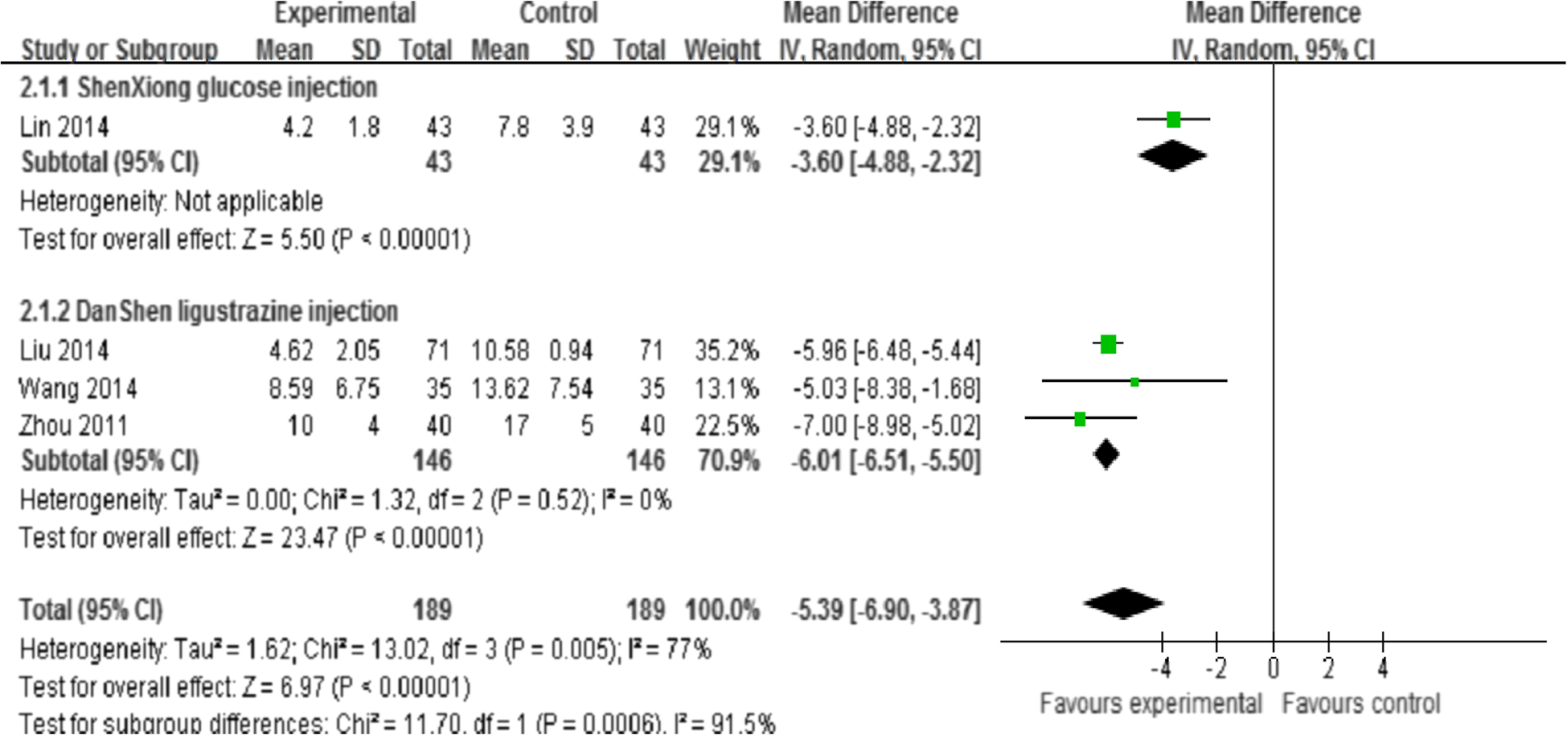
Subgroup analysis. ShenXiong glucose injection vs. DanShen ligustrazine injection; Outcome: Improvement of neurological deficit at 2 weeks follow up

Four trials [30, 32, 36, 37] assessed improvement of neurological function deficit at 14 days on treatment, as there was significant heterogeneity among studies (Chi- squared *P* = 0.005, I^2^ = 77 %), which was possibly due to the differences in outcome measurements used and types of treatment. A subgroup analysis based on the treatment type (ShenXiong glucose injection [36] and Danshen ligustrazine injection [30, 32, 37]) indicated that there were significant difference in improvement of neurological function deficit at 14 days in the ShenXiong glucose injection compared with control group (MD = −3.60, 95 % CI: −4.88, −2.32), as well as in the Danshen ligustrazine injection group compared with control group (MD = −6.01, 95 % CI: −6.51, −5.50) (Fig. 5).

Four studies [31–33, 35] also assessed the improvement of neurological function deficit at 28 days. A pooled analysis of these studies found a significant difference in the neurological function deficit scores at 28 days between the treatment groups (MD = −5.16, 95 % CI: −6.49, −3.83), low level of heterogeneity was detected among the studies (*P* = 0.31, I^2^ = 16 %) (Fig. 4).

### Response rate

**Fig. 6:**
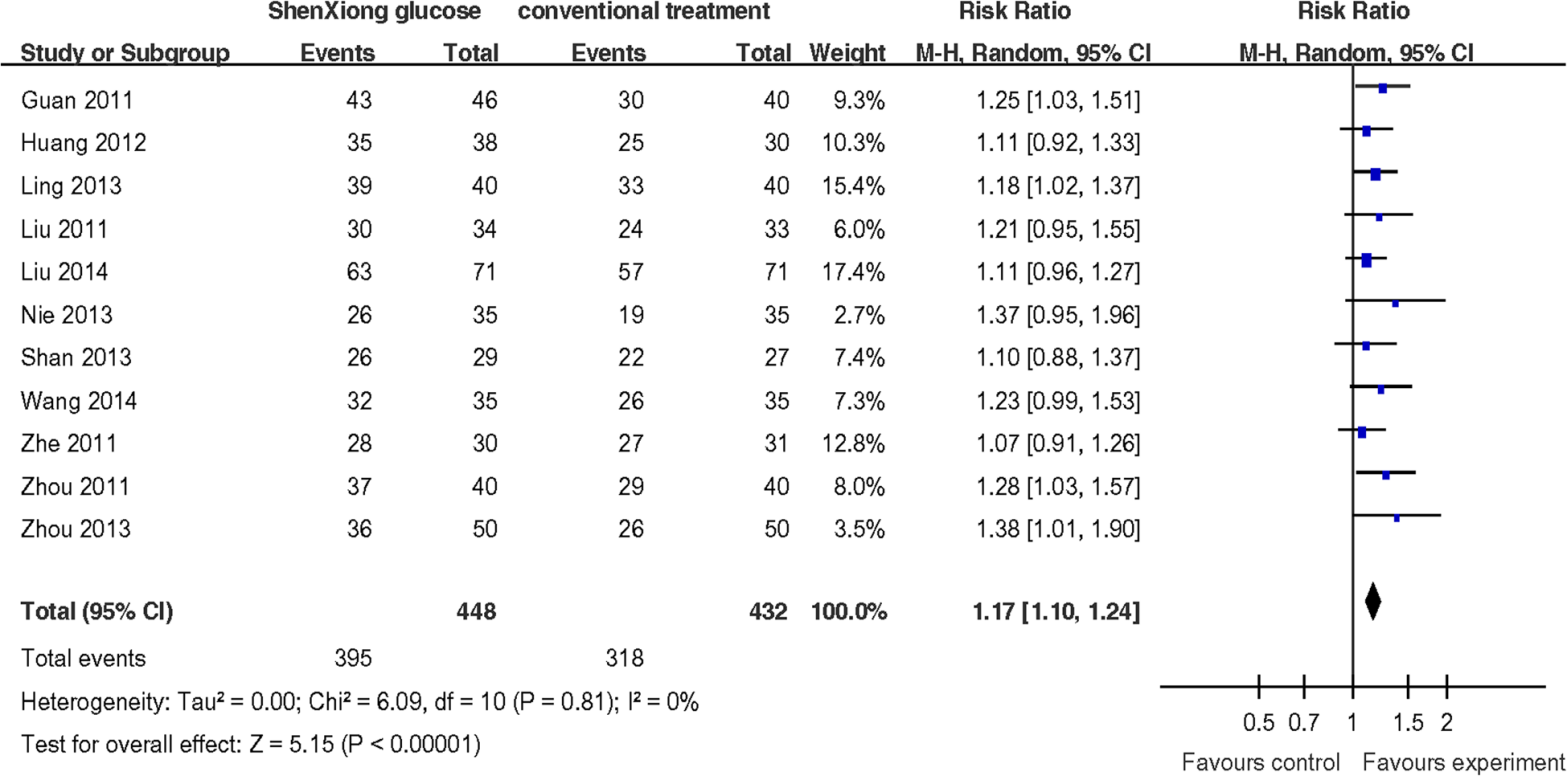
A random effects Meta-analysis on response rate of ShenXiong glucose injection

The response rate taken as a measure of clinical effects was presented in eleven trials involving of 884 patients, there was no statistically significant heterogeneity in those studies (*P* = 0.81, I^2^ = 0 %). Meta-analysis of 11 trials showed that ShenXiong glucose injection had significantly higher response rate than that of control group (RR = 1.17, 95 % CI: 1.10, 1.24) (Fig. 6).

### Safety evaluation

Six trials clearly reported that there were no adverse events during the trials, while the left studies failed to report whether adverse events happening or not. The meta-analysis didn’t perform due to numerical data for the outcomes of interest were unavailable.

### Publication bias

**Fig. 7:**
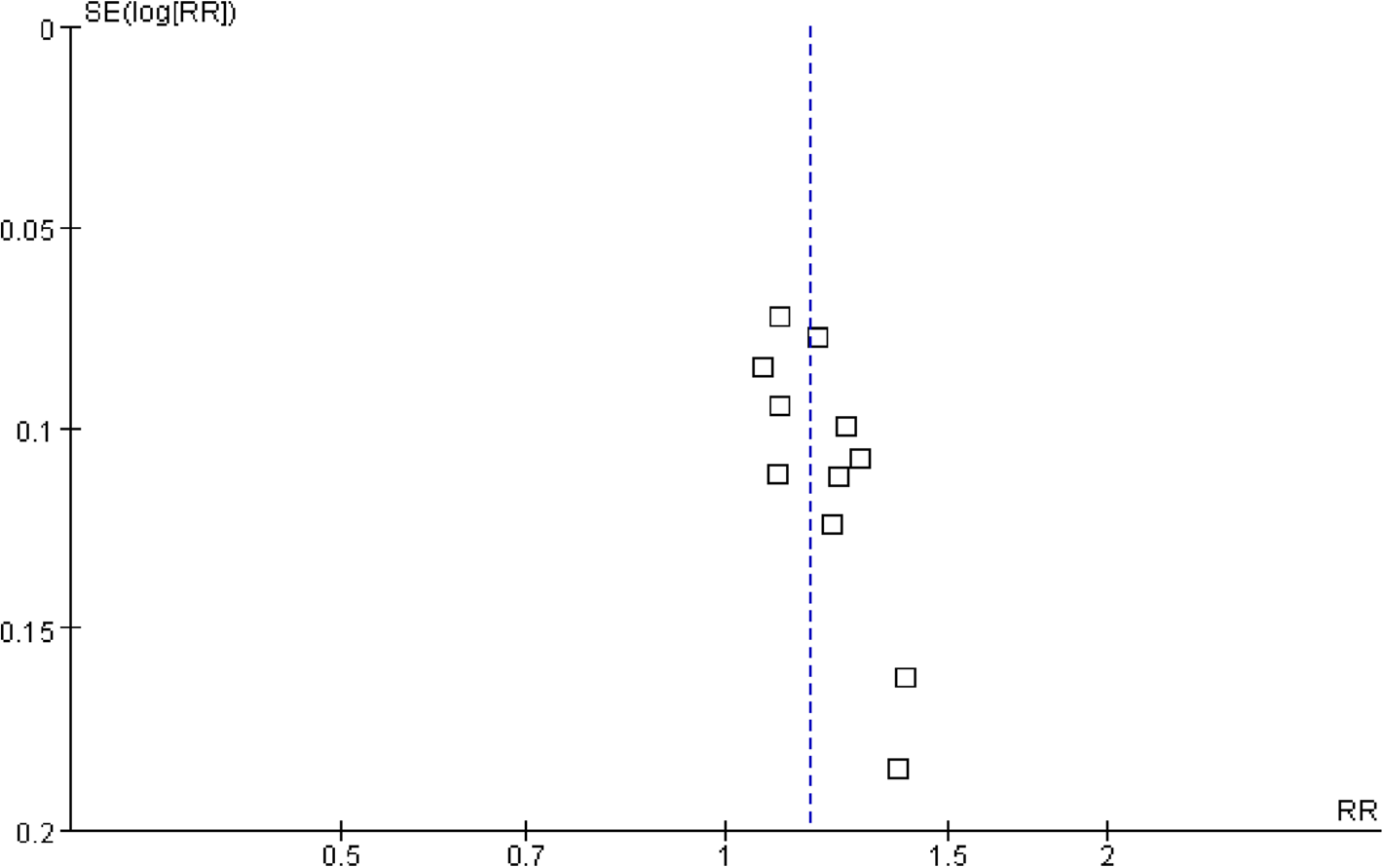
Funnel plot analysis. Funnel plot analysis on response rate of the 11 trials comparing ShenXiong glucose injection with conventional treatment

A funnel plot analysis on response rate of 11 trials was generated to explore the potential publication bias, and it present asymmetric trend, indicated that potential publication bias may exist in this review (Fig. 7).

### The quality of evidence by using GRADE

In addition, we applied GRADE to grade the quality of available evidence, regarding to this GRADE approach, all 12 RCTs started out as high-quality evidence. Of those, seven trials [30–36] reported the improvement of neurological function deficit score (NFDS), the quality of the evidence was downgraded as moderate due to inconsistency and possible publication bias; Barthel index score was taken as critical outcome measure in five RCTs [31–35], the low quality was graded due to imprecision, inconsistency, and significant heterogeneity; eleven studies evaluated clinical effects by the use of response rate, and the evidence was set as moderate quality, publication bias and the lack of directness were the main considerations for downgrading (Table 3 and Additional file 1).

**Table 3:** ShenXiong glucose injection plus conventional treatment compared to conventional treatment for acute ischemic stroke

Outcomes	Illustrative comparative risks^a^ (95 % CI)		Relative effect (95 % CI)	No of Participants (studies)	Quality of the evidence (GRADE)	Comments
	Assumed risk	Corresponding risk				
	Conventional treatment	ShenXiong glucose plus conventional treatment				
Death	none	none	none	none	none	CRITICAL
NFDS Follow-up: 1–4 weeks	none	The mean NFDS in the intervention groups was -4.70 lower (−6.04 to −3.36 lower)	-	821 (7 studies)	⊕ ⊕ ⊕⊝ moderate	CRITICAL
NFDS - 1 week	none	The mean NFDS - 1 week in the intervention groups was −2.37 lower (−5.44 lower to 0.7 higher)	-	156 (2 studies)	⊕ ⊕ ⊝⊝ low [1, 2]	CRITICAL
NFDS - 2 weeks	none	The mean NFDS - 2 weeks in the intervention groups was −5.39 lower (−6.90 to −3.87 lower)	-	378 (4 studies)	⊕ ⊕ ⊕⊝ moderate [3–6]	CRITICAL
NFDS - 4 weeks	none	The mean NFDS - 4 weeks in the intervention groups was −5.16 lower (−6.49 to −3.83 lower)	-	287 (4 studies)	⊕ ⊕ ⊕ ⊕ high [7, 8]	CRITICAL
Barthel score	none	The mean Barthel score in the intervention groups was 19.98 higher (10.17 to 29.79 higher)	-	477 (5 studies)	⊕ ⊕ ⊝⊝ low	CRITICAL
Barthel score - 1 week Follow-up: mean 1 weeks	none	The mean Barthel score - 1 week in the intervention groups was 4.65 higher (−1.56 lower to 10.86 higher)	-	70 (1 study)	⊕ ⊕ ⊕⊝ moderate [2]	CRITICAL
Barthel score - 2 weeks Follow-up: mean 2 weeks	none	The mean Barthel score - 2 weeks in the intervention groups was 12.14 higher (0.47 to 23.82 higher)	-	150 (2 studies)	⊕ ⊕ ⊝⊝ low [2, 9]	CRITICAL
Barthel score - 4 weeks Follow-up: mean 4 weeks	none	The mean Barthel score - 4 weeks in the intervention groups was 28.09 higher (15.12 to 41.07 higher)	-	257 (4 studies)	⊕ ⊕ ⊝⊝ low [10, 11]	CRITICAL
Response rate Follow-up: mean 4 weeks	Study population		RR 1.17 (1.1 to 1.24)	880 (11 studies)	⊕ ⊕ ⊕⊝ moderate [10]	IMPORTANT
	736 per 1000	861 per 1000 (810 to 913)				
	Moderate					
	750 per 1000	878 per 1000 (825 to 930)				

Patient or population: patients with acute ischemic stroke Settings: inpatients, Intervention: ShenXiong glucose injection plus conventional treatment, Comparison: conventional treatment

^a^The basis for the assumed risk (e.g. the median control group risk across studies) is provided in footnotes. The corresponding risk (and its 95 % confidence interval) is based on the assumed risk in the comparison group and the relative effect of the intervention (and its 95 % CI)

*CI* Confidence interval

*RR* Risk ratio

GRADE Working Group grades of evidence

(⊕⊕⊕⊕) High quality: Further research is very unlikely to change our confidence in the estimate of effect

(⊕ ⊕ ⊕⊝) Moderate quality: Further research is likely to have an important impact on our confidence in the estimate of effect and may change the estimate

(⊕ ⊕ ⊝⊝) Low quality: Further research is very likely to have an important impact on our confidence in the estimate of effect and is likely to change the estimate

(⊕⊝⊝⊝) Very low quality: We are very uncertain about the estimate

[1] Heterogeneity test: *P* = 0.10, I^2^ = 63 %. Obvious inconsistency exists in only two studies

[2] Imprecision (large width of CI). Sample sizes and number of events less than the number of patients generated by a conventional sample size calculation for a single adequately powered trial

[3] As the random sequence generation was not clearly described in only two of the trials, the overall risk of bias is not serious

[4] Heterogeneity test: *P* = 0.005; I^2^ = 77 %. The available data do not permit an interpretation as to the reason for some of the inconsistencies that were found. Such inconsistency is also a basis for downgrading the level of evidence in support of ShenXiong glucose injection for AIS

[5] Although the sample sizes and number of events (*n* = 292) less than the number of patients generated by a conventional sample size (*n* = 300) calculation for a single adequately powered trial, the change of our confidence for this outcome not serious, thus not downgrade

[6] The funnel plot present asymmetric trend

[7] Although the sample sizes and number of events (*n* = 287) less than the number of patients generated by a conventional sample size (*n* = 300) calculation for a single adequately powered trial, the overall imprecision for this outcome is not serious, thus not to downgrade the quality of evidence for imprecision

[8] Heterogeneity: *P* = 0.02; I^2^ = 82 %. The available data do not permit an interpretation as to the reason for some of the inconsistencies that were found. Such inconsistency is also a basis for downgrading the level of evidence

[9] Heterogeneity: *P* < 0.00001; I^2^ = 90 %. The available data do not permit an interpretation as to the reason for some of the inconsistencies that were found. Such inconsistency is also a basis for downgrading the level of evidence

[10] The funnel plot present asymmetric trend on clinical efficacy, and the risk of publication bias may exist in somewhat

[11] Heterogeneity: (*P* = 0.39); I^2^ = 0 %, mild inconsistency maybe exists in 4 studies

## Discussion

### Summary of main findings

This review was done in accordance with the methods of a Cochrane systematic review. We identified and included 12 trials involving a total of 974 participants. This systematic review has shown that ShenXiong glucose injection given as a treatment in the acute stage of ischemic stroke significantly effect on improving activities of daily living function at 7 days, 14 days and 28 days whilst on treatment, but a meta-analysis of this outcome using data from the five studies was not performed due to obvious heterogeneity among the study estimates. Significant improvements on activities of daily living function were observed from individual trials’ results. We performed subgroup analysis based on the type of treatment (ShenXiong glucose injection and Danshen ligustrazine injection). No clear difference in Barthel index score at 28 days was present between ShenXiong glucose injection trial and Danshen ligustrazine injection trial. None of the included studies reported the pre-specified primary outcome of death during the follow-up period, it may be explained that authors attempted to emphasize short-term effect of ShenXiong glucose injection, or that there were no deaths during the short-term follow up period. Furthermore, pooled results showed ShenXiong glucose injection had significant effect on the improvement of neurological function deficit at 14-day and 28-day whilst on treatment. But this results should be interpreted with caution because of severe heterogeneity was seen among the review. We performed subgroup analysis based on the type of treatment (ShenXiong glucose injection and Danshen ligustrazine injection) to explore the reasons for large heterogeneity. It showed no evidence of difference in efficacy between ShenXiong glucose injection and Danshen ligustrazine injection. There is evidence that ShenXiong glucose injection has clinical effects, such as higher response rate, this result is consistent with the improvement of neurological function deficit. Lastly, only six trials reported that no adverse events were observed during the trials period, while the left studies failed to report whether adverse events happening or not. We cannot draw a conclusion on the safety of ShenXiong glucose injection. There is currently not enough evidence to determine whether the use of ShenXiong glucose injection in acute ischemic stroke would result in any harmful effects.

After applying GRADE approach for this systematic review, although rating down for the risk of bias was not required, Unexplained heterogeneity was found within included studies, while the available data did not permit an interpretation as to the reason for some of the inconsistencies we found, such inconsistency is also a basis for downgrading the level of evidence to support ShenXiong injection for AIS patients. In addition, regarding the two important outcomes, the total number of events or patients does not exceed the OIS (Optimal Information Size), therefore, rating down for imprecision was warranted [38]. By the way, the quality of evidence was not reduced in concerning indirectness. And finally, despite the fact that none of the included studies were found by pharmaceutical industry, the funnel plot present asymmetric trend on clinical efficacy, and the risk of publication bias may exist in somewhat.

Therefore, our GRADE approach leads to the decision that ShenXiong glucose injection for a individual patient with acute ischemic stroke should be approached cautiously, patients‘values and preferences should be considered and allow them to participate in the medical decision making process.

### The limitations of the twelve trials assessed to be of inferior methodological quality were as follows

1. Only two trials using a random method to divide the groups, the remaining 10 trials reported ‘randomly allocating’ participants but the method of randomisation was not described. 2. None of the included trials reported allocation concealment, and whether a blinding method used or not within twelve trials is unclear, increasing the risk of selection or performance bias happening. 3. In addition, all included studies were conducted in China and were published in Chinese journals. A funnel plot analysis also indicated asymmetry trend for the outcome of response rate, publication bias maybe exist, although we attempt to perform comprehensive search as well as supplementary search for both published and unpublished literature. 4. Another limitation of the review relate to the over-proportion of small trials to be included, may cause imprecision and inconsistency of effect size among those trials. Although the estimate of effect on the outcomes is significant statistically, the observed effects could also simply be due to bias rather than to a biological effect of ShenXiong glucose injection.

In addition, the usefulness of evidence was limited due to the following reasons,. Firstly, the follow-up time was insufficient to evaluate the long term effects of ShenXiong glucose injection, in the 12 trials, follow-up time ranged from 7 days (1 week) to 28 days (4 week) after the start of treatment. Secondly, the reporting on adverse effect was poor in most of included trials, it is highly implausible that no adverse event happen among 494 patients, the safety of ShenXiong injection is still inconclusive. And additional limitations in this review include the diversity of outcome measure used by the included studies, and the limited duration of follow up (generally no more than 2 months).

In summary, the overall quality of evidence was judged to be low to moderate due to important limitations exist in study design, inconsistency, lack of directness, imprecision and possible publication bias. The result of published intervention trials are therefore of insufficient quality to enable evidence-based recommendations to be developed for clinical practice in acute ischemic stroke. This review systematically describes current evidence from latitudinal and longitudinal studies on ShenXiong glucose injection and identifies methodological aspects to be improved in further research. This methodological improvement is pivotal for the assessment of the body of evidence on ShenXiong glucose injection treatment. High-quality evidence from methodologically via double-blinded randomized controlled trials are required to provide further support for administration of ShenXiong glucose injection for patients with acute ischemic stroke.

## Conclusion

### Implications for practice

The results of this review provided evidence to support that the use of ShenXiong glucose injection may improve rehabilitation for patients with acute ischemic stroke, however, as the GRADE approach indicated low to moderate quality of available evidence as well as insufficient information about harm and patients preference, the recommendations for ShenXiong glucose injection for treating patients with acute ischemic stroke were not provided in this context.

### Implications for research

Further RCTs on ShenXiong glucose injection are needed and should be reported according to the CONSORT (Consolidated Standards for Reporting Trials, CONSORT) statement. High quality on the reporting of trials is strongly required.

## Additional file

Additional file 1:
**The PRISMA checklist.** (DOC 62 kb)
